# Potential Haptic Perceptual Dimensionality of Rendered Compliance

**DOI:** 10.3390/biomimetics8010064

**Published:** 2023-02-04

**Authors:** Zhiyu Shao, Jingwei Li, Wanlu Feng, Hongru Tang

**Affiliations:** School of Electrical, Energy and Power Engineering, Yangzhou University, No. 88 South University Road, Yangzhou 225009, China

**Keywords:** compliance perception, haptic perceptual dimensions, multidimensional scaling, haptic interface, human–computer interaction

## Abstract

Studies have proven that humans perceive haptic textures through different perceptual dimensions, such as rough/smooth and soft/hard, which provide useful guidance in the design of haptic devices. However, few of these have focused on the perception of compliance, which is another important perceptual property in haptic interfaces. This research was conducted to investigate the potential basic perceptual dimensions of the rendered compliance and quantify the effects of the simulation parameters. Two perceptual experiments were designed based on 27 stimuli samples generated by a 3-DOF haptic feedback device. Subjects were asked to describe these stimuli using adjectives, classify the samples, and rate them according to corresponding adjective labels. Multi-dimensional scaling (MDS) methods were then used to project adjective ratings into 2D and 3D perception spaces. According to the results, hardness and viscosity are considered two basic perceptual dimensions of the rendered compliance, while crispness can be regarded as a subsidiary perceptual dimension. Then, the relations between simulation parameters and perceptual feelings were analyzed by the regression analysis. This paper may provide a better understanding of the compliance perception mechanism and useful guidance for the improvement of rendering algorithms and devices for haptic human–computer interaction.

## 1. Introduction

A haptic interface is an important topic in human–computer interaction. Haptic rendering algorithms and devices can provide feedback on common haptic feelings such as texture, compliance and shape. High-quality haptic feedback can enhance the immersion and realism in human–computer interaction, which has been widely used in many areas such as virtual surgery [1], education for the blind [2] and virtual reality games [3].

Simulating the feelings of compliance is a very important part of haptic interaction. Force feedback from compliant objects could help users more easily determine the operating position and operate more precisely. Prior to simulating real compliance, it is a fundamental task to study the human haptic perception mechanism of compliance perception, which could provide lots of guidance for the design and improvement of rendering algorithms and devices.

According to previous studies on the compliance perception, people can perceive compliance in many different ways such as touching, tapping one’s fingers or with a tool [4,5,6]. The perception of compliance is described as the subjective assessment of the amount of deformation on an object in response to an applied force, which is usually measured by stiffness or Young’s modulus [7,8,9]. However, most of the compliant objects in our daily life have more than just one feeling besides stiffness, such as friction and viscosity. In most cases, compliance is a comprehensive description for this feeling [10]. The human perceptual system perceives, transforms and processes these cues of haptic stimuli through different perceptual channels and dimensions. Thus, subjective feelings of compliance are influenced by a series of factors such as contact modes, rendering algorithm and interactive environments [11,12,13,14].

The haptic perception of virtual compliant objects is different from real ones due to the exploratory modality [15]. In the real environment, users can perceive the objects either by directly pressing or tapping with bare fingers, or with a tool. However, force feedback devices are indispensable in most cases of compliance perception in the virtual environment, as users can only perceive compliance through a probe. This may lead to the disappearance of some key cutaneous information which is mainly conducted through the skin deformation of fingers. Many experiments and analyses have been performed to research the difference in recent years. Bergmann Tiest and Kappers [8] determined that the information that humans use for the compliance perception consists of 90% cutaneous information and 10% kinesthetic information by comparing the discrimination thresholds with and without cutaneous information. This explained the principles of many experimental results. LaMotte [16] found that the cutaneous information could be transmitted to human fingers when perceiving compliance using a rigid tool. Hauser [17] also proved that force–rate cues play more important roles in discriminating compliances than the skin. Thus, in the simulation and perception of virtual rendering compliance, it is important to make sure that the probe is set as rigid to conduct the force-rate cues as much as possible.

The differences caused by the real and virtual perceptual environment are also reflected in the perceptual dimensions. For example, in the process of quantifying the fidelity of the haptic rendering of deformable objects, Leškowský [18] found that a one-dimensional space was sufficient to describe real samples but two dimensions were required when virtual samples were added. The conclusion indicated that virtual rendering compliance might have different perceptual dimensions with real samples. Thus, the study of the potential perceptual dimensions of rendering compliance is a foundational work for follow-up studies. Rosenberg [19] analyzed the feelings of compliance from a perceptual point of view. The conclusions indicated that perceptual compliance could be decomposed into three perceptual qualities: the crispness of initial contact, the hardness of surface rigidity, and the cleanness of final release. Their decomposition was mainly conducted according to the perception stages in the interaction process rather than different perceptual channels and dimensions. Conclusions from Üstün [20] indeed showed the dissociation of perceptual dimensions. Cavdan [21] also revealed how the perceptual dimensions were influenced by the material and perceptual task. However, most of the studies focused on the number of dimensions and had few discussions on the specific meanings and the relationships of the dimensions. Furthermore, the mapping relations between the controlling parameters and perceptual dimensions are also very important in the design and improvement of rendering hardware and algorithms.

In the real environment, haptic feelings of compliance are mainly affected by the materials of the samples. However, in the virtual environment, the perceptual feelings of compliance are mainly affected by rendering algorithms. Generally speaking, a model of stiffness, damping and inertia can cover most compliant objects in our daily life. Furthermore, in the process of actual rendering, different combinations of these parameters will generate different feedback. Studies [22] have found that the perceived magnitude of compliance was closely related to the sequence of occurrence of force and deformation. Jacinto [23] investigated how the discrimination ability would be affected by including a delay and damping in the interaction of the user with the virtual tissues. In 2000, Lawrence [24] proposed a new performance metric named rate-hardness to measure the perceived compliance instead of the traditional ratio of force and displacement in the virtual environment. Based on it, Han [25] presented the extended rate-hardness as a measure for perceived compliance, which covered a larger class of rendering algorithms and applications. Many studies [26,27,28] found that it was an effective way to add damping into the compliance rendering system through the controller to ensure a higher rendering quality. Nuño, Basañez and Ortega [29] also showed that one effective method to guarantee stability was to make sure that the ratio of adding damping by the controller and the proportional controller gain was bounded from below. Beek [30] investigated the effect of damping on the perceived hardness of an object and the results showed that perceived compliance was influenced by damping in a task-specific way. Most of these studies qualitatively discussed controlling algorithms and hardware rather than discussing the perceptual characteristics or relationships between controlling parameters and the perceptual dimensions of simulated compliance.

In the area of haptic texture perception, many studies have demonstrated that the perception of textures consisted of different psychophysical dimensions [31,32,33]. Okamoto [34,35] reviewed those methods and results and concluded that haptic textures are composed of three prominent psychophysical dimensions which are perceived as roughness/smoothness, hardness/softness, and coldness/warmness. These provided a lot of inspiration for the research in this paper. As such, the purpose of this research was to determine whether there are also basic psychophysical dimensions that constitute the comprehensive perception of compliance and analyze the relationships between them. We investigated the perceptual characteristics of compliance through a haptic rendering device in psychophysical experiments. Perceptual feelings of compliance were rated by subjects while physical parameters that probably affect the results were controlled. Relations between physical parameters and subjective feelings were analyzed by the MDS method and regression analysis in the process. The results in this paper may contribute to a better understanding of the generation of compliance perception and the factors that influence the perception.

## 2. System and Methods

### 2.1. Methodology for Specifying Perceptual Dimensions

The aim of investigating the dimensionality of compliance is to decompose the comprehensive feelings to basic cues that can be clearly described and relate physical parameters to subjective description. Based on lots of works on different kinds of haptic perception dimensions [36,37,38], the conclusion can be obtained that there are usually two steps to determine the perceptual dimensions for one kind of haptic perception. Okamoto [34,35] summed up the common methods for these two steps:

The first step is the collection of subjective data for the stimuli. Typical subjective data are the perceptual ratings of test samples using adjective labels and perceptual similarities between the stimuli. The second step is the multivariate analysis for subjective data. As such, there is a pilot experiment and a main experiment in the research.

The pilot experiment was designed for the collection of adjective labels and the similarity judgment. The adjective labels were collected to help in interpreting and determining the possible psychophysical dimensions from the MDS solutions. The similarity judgment was used to measure the difference between samples and generate the dissimilarity matrix for the MDS method. The main experiment was designed for the adjective ratings for the samples and the adjective scores would be mapped into the perceptual spaces to show and explain the psychophysical meanings of the dimensions. Here, the MDS is a method to show the dissimilarities between pairs of samples using distances between corresponding points in a low-dimension perceptual space [39]. Data points representing different test samples can be displayed and observed according to the graphical display of the correlations provided by MDS. Psychological representation information of the stimuli can be provided by the MDS solution spaces, including the configuration of the test samples, the dimensionality of the perception, and the interpretation and relations of the dimensions. In general, adjective labels that are as orthogonal as possible in the MDS perceptual space are proper dimensions for haptic compliance perception, which is an important purpose of the analysis in this paper.

### 2.2. Experiment Design

#### 2.2.1. Participants

Fifteen participants in total recruited from Yangzhou University took part in the experiments: all of them (9 males and 6 females, with an average age of 25.8 years) participated in the pilot experiment, whilst 8 (5 males and 3 females, with a mean age of 24.3 years old) of them participated in the main experiment. All subjects were right-handed and have no known cutaneous or kinesthetic problems. All of them received written instructions and signed an informed consent form before the experiments. In addition, they were compensated for taking part in the experiments.

#### 2.2.2. Apparatus

**Figure 1:**
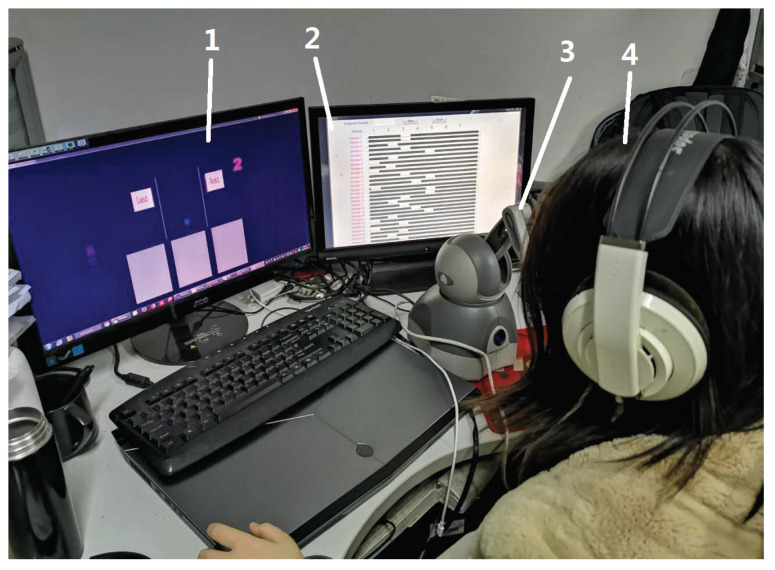
Experimental platform. 1: Compliance rendering scene based on Openhaptic SDK. 2: MATLAB GUI for data record. 3: Haptic interaction device. 4: The participant.

The experimental system consists of three parts: the haptic rendering device, the compliance rendering scenes and a GUI for data collection. Subjects interacted with haptic rendering scenes on the computer through the haptic device and made their responses in the GUI. The Geomagic Touch device was used to generate the force rendering feedback of compliance in this paper. The maximum force that can be generated by the device Touch is 3.3 N in a 3D space of 160 mm × 120 mm × 70 mm. The vertical direction (Y axis) was the main haptic rendering direction in the rendering because it has the maximum stiffness feedback of 2.31 N/mm with a resolution of 0.055 mm and is easier to perceive for participants compared with other directions. The rendering of compliance was performed on VS 2010 with the open-haptic SDK supported by the force dimension and the GUI was performed with MATLAB on a computer (as seen in Figure 1).

#### 2.2.3. Stimuli and Set-Up

Based on previous studies mentioned in the introduction, it can be concluded that the compliance perception is a combination of force, deformation and cutaneous cues changing with time. As mentioned in the introduction section, in a haptic rendering system for compliance, force and deformation information is usually affected by stiffness and damping coefficients. Cutaneous cues changing with time is influenced by initial contact speed. As such, the physical parameters of the stiffness coefficient, damping coefficient and exploration speed were selected as controlled independent variables in this paper. More types and levels of parameters mean more samples, more complex experimental procedures and a longer experimental time, which may be too hard for the participants to make the right choices. According to the restriction of rendering capabilities, the limit of the subject discrimination ability and prevent an excessive number of experimental rounds, 3 levels of stiffness, 3 levels of damping and 3 levels of exploration speed were considered in the experiments (as listed in Table 1), which cover a reasonable range of the compliance rendering.

**Figure 2:**
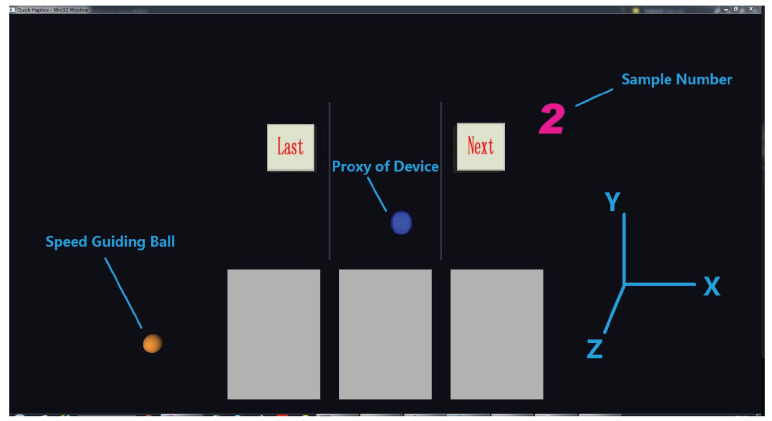
Compliance rendering environment. The ball in the middle is the proxy of the haptic rendering device, the ball on the left is the speed guiding ball and stimuli are presented in the gray blocks. The figure on the upper right is the number of the present sample.

Each rendering sample present to the subject has its corresponding levels of stiffness, damping and exploration speed, so there are a total of 27 samples in this paper. As shown in Figure 1 and Figure 2, the stimuli of compliance were presented in the gray blocks in the lower part of the screen. The blue ball is the proxy of the haptic rendering device in the virtual environment. When participants moved the Geomagic Touch probe up and down in the real world, the proxy would move up and down (along the Y axis) correspondingly. When the blue ball moved into the gray blocks, participants would feel the specific feedback generated by the device according to the preset stiffness and damping coefficient levels. It is worth noting that, all the blocks would not have a visual deformation throughout the experiments to avoid the influence of visual channel on haptic feelings. The levels of the exploration speed of participants were controlled by speed guiding through the orange ball. The orange speed guiding ball would move up and down at a constant speed (one of the 3 speed levels) and the participants were asked to move the proxy on the screen following it at a speed as close as to the orange one.

#### 2.2.4. Procedure

Participants sat in front of the experimental table wearing noise-canceling headphones with the rendering tasks and data collecting GUI running (seen in Figure 1). Participants were asked to operate the Geomagic Touch with their dominant hand when the experiments started, move the proxy ball following the guiding speed in the rendering environment to perceive the present rendering stimuli and then give their response in the data collecting GUI. There were two experiments that need to be completed by the participants: similarity judgment (the pilot experiment) and adjective rating (the main experiment). In each experiment, 27 samples would be present among the participants in a random order in the gray blocks, in case the participants make a choice or judgment by experience or guess. Participants were allowed to move the proxy up and down to perceive the compliance until they were sufficiently satisfied to make a decision or judgment. The participants could change the present sample to any one they want by clicking the corresponding buttons (last or next, shown in Figure 2) on the screen. There is no time limit for each perception but they were asked to follow the given exploration speed on the screen. Participants who were unfamiliar with the experimental processes or could not follow the guiding ball well could practice as many times as they wanted before the experiments. The pilot experiment took approximately 30 min and the main experiment took approximately 90 min per participant on average. The participants could take a break at any time during the experiments.

##### The Pilot Experiment

**Figure 3:**
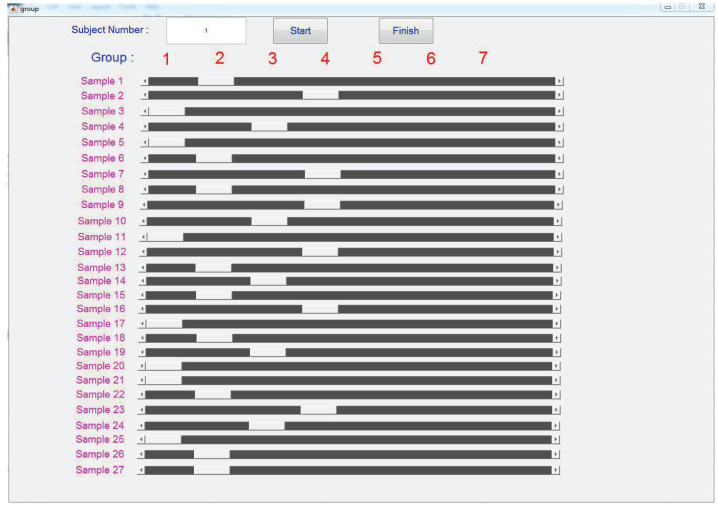
MATLAB data collection GUI for the pilot experiment. Sliders in the same column indicating their corresponding samples are classified into the same group.

In the pilot experiment, the participants were asked to finish the similarity judgment. Specifically, the participants perceived all 27 stimuli, described their feelings regarding the samples using as many adjective labels as possible and then place the samples which had the similar feelings into the same group. As shown in Figure 2 and Figure 3, all 27 samples would appear in the middle gray blocks in turn (the left and right gray blocks were not used in the pilot experiment). The participants would first go through all 27 samples and described their feelings about the samples using any adjectives they like. For example, hard, stick, rough, elastic, comfortable, and so on. Adjective labels would be selected from these adjectives in following data analysis. Then, the participants were asked to perceive the samples one by one and classify them into at least 3 and at most 7 groups. As shown in Figure 3, if the participants thought that some of the samples had similar feelings and should be in a same group, they can move the corresponding sliders in the GUI under to the same number labels. The participants were encouraged to revisit each test stimuli and confirm their results before clicking the finish button.

##### The Main Experiment

**Figure 4:**
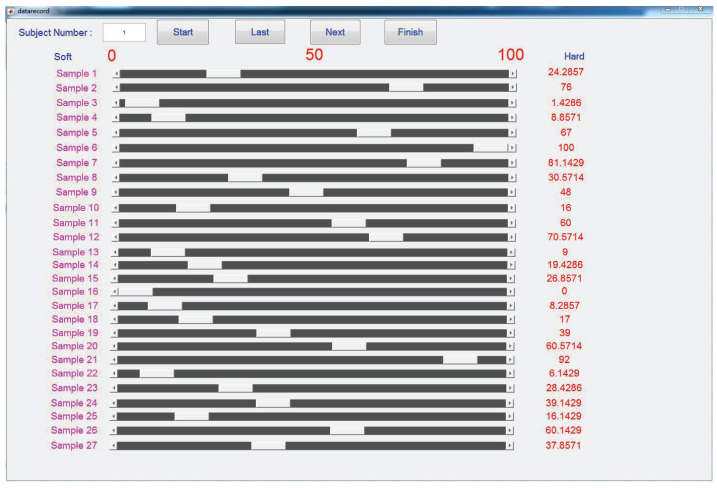
MATLAB data collection GUI for the main experiment. The positions of the slider bars were linearly mapped to scores between 0 and 100 from left to right. Participants move the sliders to rate the current adjective label.

In the main experiment, the participants were asked to complete the adjective rating. They had to rate all 27 samples focusing on the feelings described by each adjective label selected from the pilot experiment. As shown in Figure 4, the adjective labels such as hardness and stickiness that needed to be focused on by the participant would be displayed on the GUI. There were five adjective labels to be rated in this paper according to the results of the pilot experiment, which would be shown in the next section. Each sample had its corresponding slider range from 0 to 100, and the 0 meant the present sample had a minimum perceptual intensity and 100 meant that the present sample had a maximum perceptual intensity to the present adjective label among all the samples. Before rating each adjective label, the participants needed to go through all the samples and select the samples that had the minimum and maximum perceptual intensity, respectively, as scoring references, which would be displayed in the left and right gray blocks throughout the experiment. When the experiment started, the participants perceived the test samples displayed in the middle gray block and were asked to rate the feelings compared with the references along the present adjective label to be focused on. The participants were allowed to revisit all the samples before modifying or confirming their results as many times as required. When the ratings of all samples were completed, the participants could click the next button and repeat the above steps to rate the samples for the next adjective label.

## 3. Experiment Results

### 3.1. Pilot Experiments

#### 3.1.1. Adjective Labels

The participants provided the following adjectives to describe their haptic feelings of the samples: hard, soft, stick, smooth, rough, elastic, crisp, granular, and unstable. In order to simplify the analysis process while covering as many sensations as possible, the adjectives provided by the participants were classified into five adjective labels according to experience of real-world perception and previous studies [19]: hardness: the property of being rigid and resistant to pressure or deformation; viscosity: the intensity of drag force when pressing, which will not change with the deformation; roughness: or graininess, feeling of granular sensation, happening in the stage of deformation; crispness: or elasticity, the difficulty of surface deformation, mostly happening in the stage of initial contact; cleanness: the difficulty of leaving the surface, mostly happening in the stage of release stage. These adjective labels were possible dimensions that could cover the feelings of compliance, but the specific relations between them need further analysis.

#### 3.1.2. Dissimilarity Matrix

**Figure 5:**
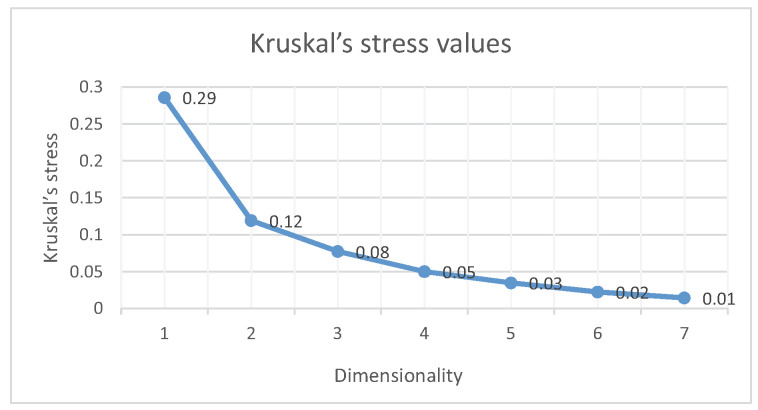
Kruskal stress values vary with the dimensionality of the MDS solution.

In the pilot experiment, the 15 participants classified the 27 samples into 4.64 ± 1.0 groups on average. A similarity matrix could be obtained from the similarity judgment. The degree of similarity between any two samples could be calculated as the number of the two samples being placed into the same group divided by the total number of participants which was 15 in the pilot experiment. As such, each sample would have their corresponding similarity values with the other 26 samples, which could form a similarity matrix. In order to analyze the experiment results by MDS method, a dissimilarity matrix could be obtained by subtracting these similarity values from 1. All the values in the dissimilarity matrix ranged from 0 to 1, and large values mean that the two samples felt dissimilar. From the process of analyzing the dissimilarity matrix using the MDS, perceptual space with different dimensions could be obtained and the Kruskal stress plot [40,41] was calculated to show whether the present number of dimensions was an optimal solution space. The stress values and their corresponding dimensions are shown in Figure 5. The values range from 0 to 1, and smaller values indicate better fitting results. As seen in Figure 5, the stress values decrease when the number of dimensionalities increases. Stress values between 0.05 and 0.6 and the dimensionality at the “elbow” point of the plot usually indicate an optimal solution [40,41]. As such, the 2D (stress = 0.12) and 3D (stress = 0.08) solutions were analyzed in this paper.

### 3.2. Main Experiment

In the adjective rating experiment, the subjects rated all the samples for each adjective label by moving the sliders left and right, whose positions were mapped linearly from 0 to 100. The final score of a certain sample for an adjective label was calculated by averaging all the scores rated by all the participants for the present adjective label. The scores of all the subjects for the five adjective labels are shown in Table 2. Larger values indicate a greater perceived intensity for the corresponding labels.

## 4. Analysis and Discussion

### 4.1. MDS Solution

**Figure 6:**
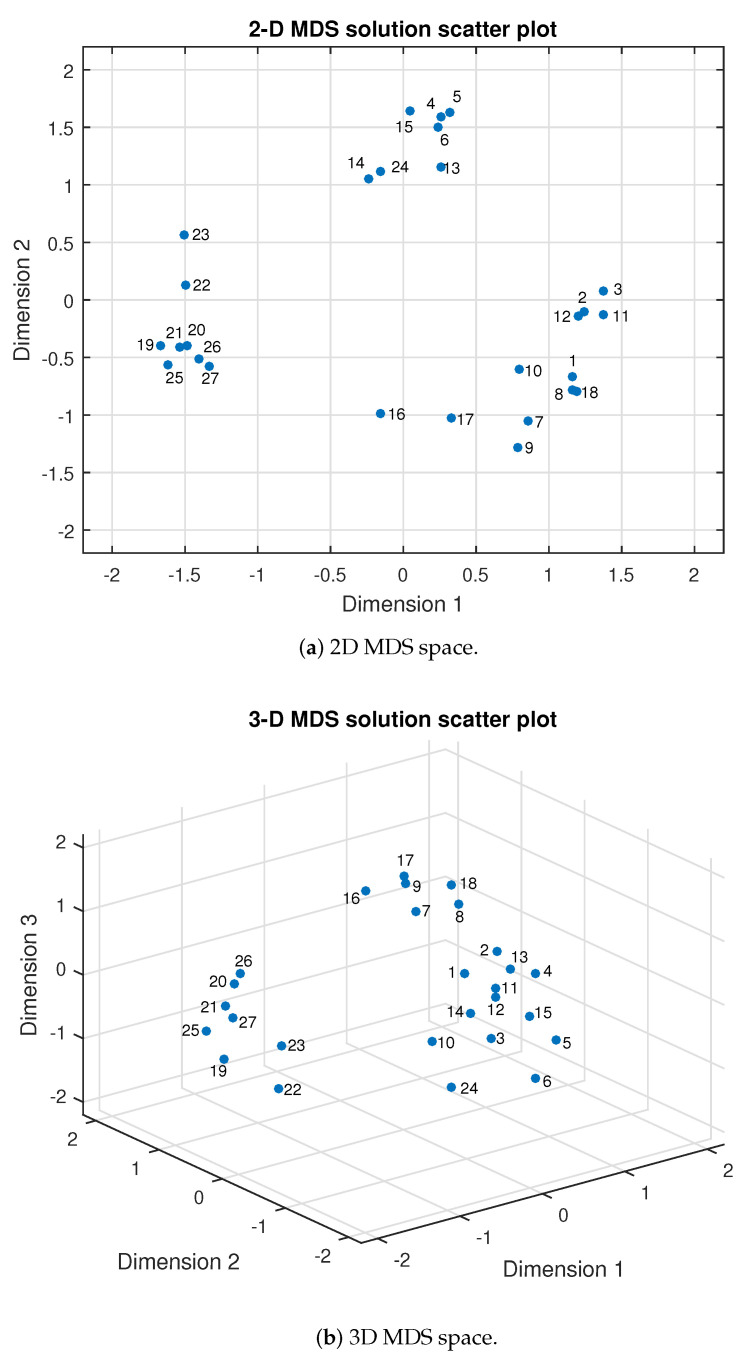
MDS solution scatter plot.

The process of the MDS method gives each of the 27 samples coordinates in 2D or 3D solution space, in which the samples can be plotted as points, and in this way, the relations between the samples are mapped into perceptual space and can be visually analyzed, as shown in Figure 6. In the 2D and 3D perception space, distances between the sample points show the difference between them and the distribution of these points would not change with the rotation of the spaces. The smaller the distance values are, the more similar feelings they have. As such, sample points that gather together in a space indicate that they have similar feelings. For example, conclusions can be obtained that samples of #19, #20, #21, #22, #23, #25, #26, and #27 have similar perceptual feelings for the participants and they are spread out on the left side in the space. Samples of #4, #5, #6, #13, #14, #15, #24 have similar perceptual feelings for the participants and they are spread out on the left side in the space. It is worth mentioning that the levels of physical parameters are not regularly changing with the number of samples because the appearance order of the samples is random for each participant. As such, in the analysis, one of the appearance orders of the samples were selected and all the other orders were adjusted to calculate the average values. As such, in Figure 6, the numbers of the samples have no special meanings and have no relations with the distribution results. What can be inferred is that there are mainly three sample concentration areas both in 2D and 3D perception space. It can be easily observed that there is a sample aggregation trend that is nearly similar in both 2D and 3D MDS perceptual spaces. As such, it is interesting and significant to analyze why these samples would gather together and how the change of physical parameter levels might influence the final distribution. Some analysis is shown in the following parts.

**Figure 7:**
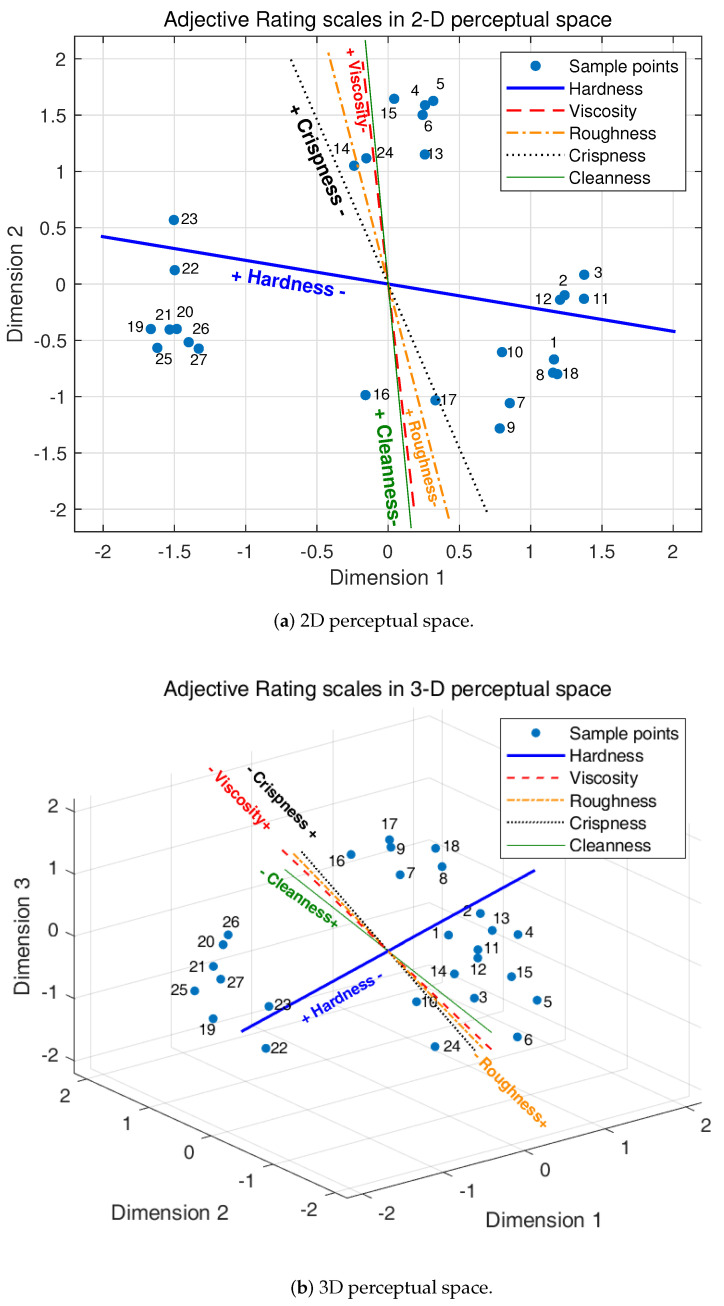
Adjective rating scales in 2D and 3D perceptual space. Different kinds of adjective labels are illustrated by different types of line. ‘+’ and ‘−’ indicate the increasing and decreasing direction of the perceived intensity along the corresponding adjective label lines.

In order to show more details of the relations between the parameter change and the distribution of the samples, average ratings of each adjective label were projected into the 2D and 3D perceptual spaces as vectors. The directions of the vectors were determined by the standardized regression coefficients [42,43] shown in Table 3 and Table 4, whilst regression coefficients with different dimensions were the direction vectors of each adjective label line. The coefficients were obtained by the multiple linear regression method with the 2D and 3D sample point coordinates as the independent variables and the adjective label ratings as the dependent variables. The standardized regression coefficients were used in the process because the sums of the squares of the standardized regression coefficients are basically proportional to the variance of the adjective scales “captured” by the MDS space [42]. The values of the determination coefficients ($R^{2}$) of all adjective labels were higher than 0.94, which means that the regression fits the data perfectly. The projection of the adjective ratings in the 2D and 3D spaces are shown in Figure 7. As seen in the figure, each adjective rating was projected as a straight line through the origin. Directions of the lines show the perceptual intensity of the corresponding adjective labels. For example, the blue line represents the adjective label of hardness. Hardness decreases from left to right and the directions are marked as + and − on the line. Other adjective labels (viscosity, roughness, crispness and cleanness) were drawn in the 2D and 3D spaces in the same way. Therefore, in the 2D perceptual space, as shown in Figure 6a, samples distributed in the left part have greater perceptual hardness and samples in the right part have smaller perceptual hardness, while samples in the upper part have greater perceptual viscosity, roughness and cleanness and samples in the lower part have smaller perceptual viscosity, roughness and cleanness. Samples in the upper left part have greater perceptual crispness and samples on the lower right part of the space have smaller perceptual crispness. The distribution regularities of these samples are the same as in the 3D MDS space as shown in Figure 6b, which are not repeatedly described in detail here.

Angles between these straight lines show the correlation between their corresponding adjective labels. Angles of 0° or 180° between two adjective labels mean that these are linear and describe the same perceptual feelings and 90° means that these two adjective labels are orthogonal and describe different perceptual feelings which would not interact with each other. The aim of this paper was to select adjective labels that are as orthogonal as possible as the basic perceptual dimensionality for compliance. Angles between these adjective labels could be calculated by the regression coefficients (direction vectors) in Table 3 and Table 4 and the results are shown in Table 5 and Table 6. It can be observed that: In both 2D and 3D perceptual space, the adjective label of hardness obviously has a different direction and large angles with the other four labels, which could be regarded as the first basic perceptual dimension for the compliance perception; hardness and cleanness have the maximum angles (73.9° and 75.8°) among the labels, which means that they have a low correlation with each other; Adjective labels of viscosity, roughness and cleanness have very small angles with each other (6.4°, 0.9°, 7.3° in 2D space, respectively, and 9.7°, 9.7°, 7.0° in 3D space, respectively), so it is obvious that they are describing the same perceptual feeling for the compliance and can be merged into one label. Here, the adjective label of “viscosity” was preserved because it is a more common and easier word to understand. As such, viscosity was selected as the second basic perceptual dimension for the compliance perception. The line of crispness was in the middle of hardness and viscosity, so crispness was kept to help understand the perceptual space and enrich the comprehensive feeling of compliance.

### 4.2. Effects of Parameters on the Compliance Perception

There are two indexes to show how the change of parameters affects the compliance perception: the perceptual difference (expressed by the dissimilarity matrix) and the perceptual intensity (expressed by the adjective-scale rating). As such, the statistical analysis was made between the physical parameters and both the dissimilarity matrix and the adjective-scale rating table. The effect of parameters on the perceptual difference of compliance is shown in Table 7. The results indicate that all of the stiffness coefficient, damping coefficient, the exploration speed and their interaction have significant effects (*p* < 0.001) on the perceptual difference. The stiffness coefficient has the greatest significance (*F* = 142.78), while the exploration speed has the least influence among the three controlled physical parameters.

The effect of parameters on the perceptual intensity of compliance is shown in Table 8, from which it can be observed that the significant effects of the stiffness coefficient on the compliance perception mainly focuses on feelings of hardness and crispness. The damping coefficient has a significant effect on all the five adjective label feelings proposed in this paper. The exploration speed has no significance on all of the adjective feelings, which is so different with its performance in the differential perception. This indicates that the influence of the exploration speed on the compliance perception is mainly on differential perception and its interaction with other physical parameters.

### 4.3. Discussion

**Figure 8:**
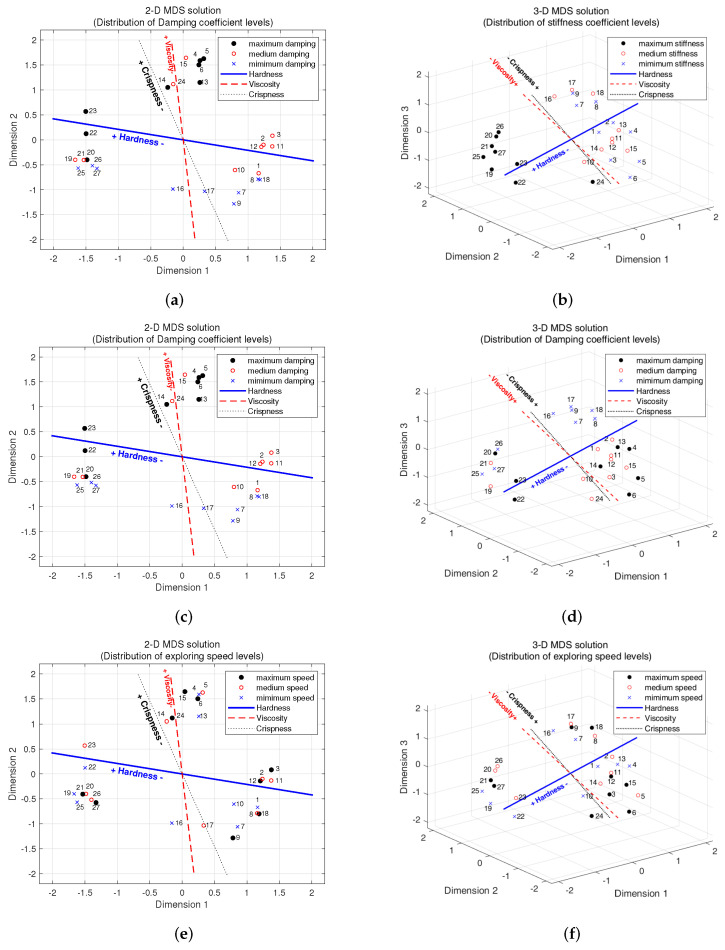
Parameter levels and adjective rating scales regressed into 2D and 3D perceptual spaces. Types of points indicate the different levels of parameters: Solid circle (‘•’) for the maximum value, cross sign (‘×’) for the minimum value and hollow circle (‘∘’) for the medium value. In (**a**,**b**), the types of points indicate the levels of stiffness. In (**c**,**d**), the types of points indicate the levels of damping. In (**e**,**f**), the types of points indicate the levels of exploration speed.

In order to determine the change rules between parameters and subjective feelings, the levels of physical parameters are expressed with different colors in the 2D and 3D perception space, respectively, with perceptual dimensions selected, shown in Figure 8.

As shown in Figure 8a,b, different colors of sample points denote the different levels of the stiffness coefficient, with black for the maximal stiffness value, blue for the minimum stiffness value and red for the medium stiffness value. It can be seen from the figure that, in both 2D and 3D perception spaces, most of the black points are concentrated on the side of the blue line that has greater perceptual intensity (left half in the 2D space), most of the red points concentrate in the middle part, while most of the blue points concentrate on the side, having smaller perceptual intensity along the blue line (right half in the 2D space), which is coincident with the direction of perceptual hardness changes. The result also confirms that the change in hardness levels has a significant effect on the perceptual hardness, as shown in Table 8. However, the distribution of the samples changing with perceptual crispness does not fit the law that well, as shown in points of #4, #5 and #6. This phenomenon also corresponds with the correlation analysis result in Table 8: the crispness adopted a smaller *F* value (*F* = 12.116) than the hardness (*F* = 239.775). The separation of the samples points away from their black, blue or red clusters (for example, #4–6 and #24 in the 2D space and #24 and #5–6 in the 3D space) may be caused by the interaction between stiffness, damping and exploration speed, which require further analysis to confirm.

As shown in Figure 8c,d, different colors of sample points stand for different levels of the damping coefficient, black for the maximal damping value, blue for the minimum damping value and red for the medium stiffness value. It can be seen from the figure that, in both 2D and 3D perception space, most of the black points concentrate on the side having greater perceptual intensity along the red and black lines (upper half in the 2D space), most of the red points concentrate in the middle part, while most of the blue points concentrate on the side having smaller perceptual intensity along the red and black lines (lower half in the 2D space), which is coincident with the direction of perceptual viscosity and crispness changes. The result also confirms that the change in damping levels has a significant effect on the perceptual viscosity (*F* = 26.829) and crispness (*F* = 174.576), as shown in Table 8. The separation of the sample points away from their black, blue or red clusters, (for example, #15 and #24 in the 2D space and #20 in the 3D space) may also be caused by the interaction between stiffness, damping and exploration speed.

As shown in Figure 8e,f, the different colors of sample points stand for the different levels of exploration speeds, black for the maximal exploration speed value, blue for the minimum exploration speed value and red for the medium exploration speed value. However, there is no similar regular pattern as there is for stiffness and damping here because the distribution of the colors seems to be irregular in both the 2D and 3D perceptual spaces. This means that the exploration speed has no significant effect on the perception of perceptual hardness, crispness and viscosity, which also corresponds with the regression analysis results: all the *p* values of the five adjective labels for the exploration speed is larger than 0.01.

In order to see how the interaction of the physical parameters affects the perception results, the quantitative analysis of the relationship between the stimulus parameters and the perceptual adjective labels was also conducted in this paper through the ANOVA method. A multiple regression analysis was used to obtain the equations which were equivalent to an input–output model. The levels of physical parameters and their interaction were considered as the independent variables while the adjective ratings of the five labels were analyzed as the dependent variables. The standardized partial regression coefficients were used in the procedure because they could eliminate the effects of the units of the physical parameters and reflect the contributions of the independent variables to the dependent variables. The results of the regression analysis are as follows:

(1) $$\left\{\begin{array}{c} H(s)=0.930k+0.311b\\ V(s)=0.917b\\ C(s)=0.938b+0.249kv \end{array}\right.$$

where the H(s), V(s) and C(s) are the hardness, viscosity and crispness, respectively. *k*, *b* and *v* are the stiffness coefficient, damping coefficient and exploration speed, respectively. The $R^{2}$ values of the hardness, viscosity and crispness are 0.958, 0.834 and 0.937, respectively, which means that the model can explain the relations between the independent variables and dependent variables very well. Equation (1) indicates that the perceptual hardness is influenced by stiffness and damping, the perceptual viscosity is only influenced by damping and the perceptual crispness is influenced by damping and the interaction between the stiffness and exploration speed. The haptic perception feeling of compliance mainly consists in the basic perceptual dimension of the hardness and viscosity and the feeling of crispness is a useful supplement.

## 5. Conclusions

This paper investigated the perceptual dimensionality of the compliance perception based on haptic interaction devices. Two experiments were conducted with the physical parameters of the stiffness coefficient, damping coefficient and exploration speed controlled. Five adjective labels were collected from the procedure and all the participants provided the perceptual results of grouping data and the adjective ratings of the five labels for the 27 test samples. Then, the MDS analysis method was introduced to generate 2D and 3D perceptual spaces based on the dissimilarity matrix obtained from the grouping data. Adjective rating data were projected into the perceptual spaces to study the relations between the parameters and subjective feelings. Results indicate that the haptic perception of compliance is a comprehensive perception consisting of feelings such as hardness, viscosity, roughness, crispness and cleanness. Correlation analysis shows that the parameter of stiffness has significant effects on the perception of hardness and crispness, the parameter of damping has significant effects on all the five adjective labels, while the exploration speed parameter has no significant effects on all five adjective labels but still influences the perceptual result by interacting with the stiffness and damping parameter. This was also confirmed by the analysis of the 2D and 3D MDS perceptual spaces and the regression analysis between parameters and subjective feelings. The MDS solution results show that viscosity, roughness and cleanness describe the same perceptual feeling for the compliance and can be merged into one label (viscosity). Therefore, hardness and viscosity are two basic psychophysical perceptual dimensions for the compliance perception, and crispness is a useful supplement for this perception.

This paper brings out some interesting problems worthy of further study. For example, the 2D or 3D perceptual spaces generated by the MDS method are both uniformly distributed, however, whether this reflects the real tactile perception mechanism is worth further verification. Perhaps some other mapping methods such as kernel principal component analysis have better results. Furthermore, the conclusions of this paper contribute to a better understanding of the haptic perception mechanism of compliance and may provide some guidance for the design and improvement of haptic devices and algorithms in future work. The basic dimensions of rendered compliance could be used comprehensively similar to the “RGB” in color modulation to generate more realistic feedback of virtual objects. This paper summarized a basic regression model of the simulation parameters, but in the actual design of the device, the physical properties of the device such as the time delay and the stability of the motor also have a significant impact on the rendering results. This is an important research problem in future work.

## Figures and Tables

**Table 1:** Parameter levels used in the experiment.

Stiffness coefficient (N/mm)	0.05	0.10	0.15
Damping coefficient (Ns/mm)	0.001	0.003	0.005
Exploration speed (mm/s)	40	80	120

**Table 2:** Mean adjective label ratings of the 27 samples.

Sample	Hardness	Viscosity	Roughness	Crispness	Cleanness
#1	21.82	12.55	10.71	11.69	4.49
#2	25.49	35.69	20.67	33.20	41.65
#3	20.43	29.84	37.43	31.29	18.31
#4	53.55	81.00	91.61	83.90	96.04
#5	91.65	23.45	23.00	54.06	37.08
#6	90.35	63.55	75.18	89.29	67.76
#7	65.96	26.65	24.49	17.80	29.41
#8	31.06	68.24	87.33	77.51	85.92
#9	87.78	74.10	68.18	69.69	42.57
#10	75.59	42.94	38.27	50.94	37.33
#11	2.73	2.53	10.33	8.33	10.80
#12	29.31	15.02	6.57	14.12	22.04
#13	88.37	62.67	75.98	79.18	73.55
#14	31.71	46.67	34.82	39.53	42.41
#15	23.65	34.53	35.69	34.69	46.08
#16	9.37	11.96	7.18	1.04	4.82
#17	26.76	25.61	26.43	17.14	17.29
#18	19.35	50.45	13.76	30.73	32.96
#19	81.20	34.78	35.02	26.88	18.41
#20	73.06	17.43	17.16	42.27	17.16
#21	4.35	18.65	11.29	9.73	78.78
#22	36.80	80.22	86.86	79.00	88.92
#23	50.53	65.51	76.84	80.55	64.20
#24	78.69	25.14	34.71	51.55	43.84
#25	20.37	51.63	32.02	30.06	38.35
#26	27.96	54.33	62.39	64.20	68.69
#27	41.53	78.41	85.92	80.18	76.80

**Table 3:** Standardized regression coefficients for each adjective label in the 2D solution.

Dimensions	Hardness	Viscosity	Roughness	Crispness	Cleanness
1	−0.876	−0.078	−0.185	−0.301	−0.070
2	0.184	0.858	0.905	0.879	0.942

**Table 4:** Standardized regression coefficients for each adjective label in the 3D solution.

Dimensions	Hardness	Viscosity	Roughness	Crispness	Cleanness
1	−0.887	−0.065	−0.178	−0.303	−0.072
2	−0.124	−0.829	−0.898	−0.842	−0.921
3	−0.195	−0.303	−0.207	−0.247	−0.168

**Table 5:** Angles between the adjective rating scales in the 2D MDS space.

2D Space	Hardness	Viscosity	Roughness	Crispness	Cleanness
Hardness	-				
Viscosity	72.9°	-			
Roughness	66.6°	6.4°	-		
Crispness	59.2°	13.7°	7.3°	-	
Cleanness	73.9°	0.9°	7.3°	14.7°	-

**Table 6:** Angles between the adjective rating scales in the 3D MDS space.

3D Space	Hardness	Viscosity	Roughness	Crispness	Cleanness
Hardness	-				
Viscosity	74.3°	-			
Roughness	68.9°	9.7°	-		
Crispness	60.3°	15.3°	8.7°	-	
Cleanness	75.8°	9.7°	7.0°	15.8°	-

**Table 7:** The effect of parameters on the perceptual difference of compliance.

Independent Variables	*F*	*p*
Stiffness coefficient	142.783	<0.001
Damping coefficient	78.499	<0.001
Exploration speed	14.665	<0.001
Stiffness coefficient × Damping coefficient	20.491	<0.001
Stiffness coefficient × Exploration speed	8.162	<0.001
Damping coefficient × Exploration speed	7.725	<0.001
Stiffness × Damping × Speed	5.110	<0.001

**Table 8:** The effect of parameters on the perceptual intensity of compliance.

Independent Variables	Adjective Labels	*F*	*p*
Stiffness coefficient	Hardness	**239.975**	**<0.001**
	Viscosity	0.118	0.890
	Roughness	0.791	0.467
	Crispness	**12.116**	**<0.001**
	Cleanness	0.226	0.800
Damping coefficient	Hardness	**26.829**	**<0.001**
	Viscosity	**54.888**	**<0.001**
	Roughness	**103.673**	**<0.001**
	Crispness	**174.576**	**<0.001**
	Cleanness	**68.321**	**<0.001**
Exploration speed	Hardness	0.531	0.596
	Viscosity	0.312	0.735
	Roughness	0.739	0.490
	Crispness	1.630	0.221
	Cleanness	2.316	0.125

## Data Availability

The datasets generated and/or analyzed during the current study are available from the corresponding author upon reasonable request.
